# Cognitive Function and Monoamine Neurotransmission in Schizophrenia: Evidence From Positron Emission Tomography Studies

**DOI:** 10.3389/fpsyt.2018.00228

**Published:** 2018-05-29

**Authors:** Harumasa Takano

**Affiliations:** Department of Clinical Neuroimaging, Integrative Brain Imaging Center, National Center of Neurology and Psychiatry, Tokyo, Japan

**Keywords:** cognitive function, schizophrenia, positron emission tomography, monoamine, dopamine, serotonin

## Abstract

Positron emission tomography (PET) is a non-invasive imaging technique used to assess various brain functions, including cerebral blood flow, glucose metabolism, and neurotransmission, in the living human brain. In particular, neurotransmission mediated by the monoamine neurotransmitters dopamine, serotonin, and norepinephrine, has been extensively examined using PET probes, which specifically bind to the monoamine receptors and transporters. This useful tool has revealed the pathophysiology of various psychiatric disorders, including schizophrenia, and the mechanisms of action of psychotropic drugs. Because monoamines are implicated in various cognitive processes such as memory and executive functions, some PET studies have directly investigated the associations between monoamine neurotransmission and cognitive functions in healthy individuals and patients with psychiatric disorders. In this mini review, I discuss the findings of PET studies that investigated monoamine neurotransmission under resting conditions, specifically focusing on cognitive functions in patients with schizophrenia. With regard to the dopaminergic system, some studies have examined the association of dopamine D1 and D2/D3 receptors, dopamine transporters, and dopamine synthesis capacity with various cognitive functions in schizophrenia. With regard to the serotonergic system, 5-HT1A and 5-HT2A receptors have been studied in the context of cognitive functions in schizophrenia. Although relatively few PET studies have examined cognitive functions in patients with psychiatric disorders, these approaches can provide useful information on enhancing cognitive functions by administering drugs that modulate monoamine transmission. Moreover, another paradigm of techniques such as those exploring the release of neurotransmitters and further development of radiotracers for novel targets are warranted.

## Introduction

Positron emission tomography (PET) is a non-invasive imaging technique used to assess various brain functions, including cerebral blood flow, glucose metabolism, and neurotransmission, in the living human brain. In particular, neurotransmission via the monoamine neurotransmitters dopamine, serotonin, and norepinephrine, has been extensively examined using PET probes, which specifically bind to the receptors, transporters, and other target molecules of these monoamines. PET provides various aspects of neurotransmission such as presynaptic and postsynaptic functions, along with anatomical information. This useful tool has revealed aspects of the pathophysiology of various psychiatric disorders, including schizophrenia, and the mechanisms of action of psychotropic drugs since monoamines are the primary targets for antipsychotic and antidepressant drugs (1). Specifically, the differences between patients and healthy controls, associations with symptomatology and psychopathology in patients, and receptor/transporter occupancy by psychotropic drugs have been reported, albeit the results have been inconsistent among studies. Since previous animal studies and human pharmacological studies have implicated monoamines in various cognitive functions, some PET studies have directly explored the associations between monoamine neurotransmission and cognitive functions in healthy individuals and patients with psychiatric disorders.

Schizophrenia, one of the most severe and complex psychiatric disorders, with a lifetime prevalence of approximately 1% worldwide, is characterized by psychosis (positive symptoms), negative symptoms, and cognitive dysfunction (2). In recent years, cognitive impairment has been considered a core feature of this disorder (3, 4), with the largest effect sizes reported in both verbal memory and executive function (5, 6). A number of functional neuroimaging studies that measured cerebral blood flow using functional magnetic resonance imaging (fMRI) and PET have revealed a relationship between cognitive deficit and altered regional brain functions (5–9). However, not many studies have used PET to explore the relationship between monoamine neurotransmission and cognitive dysfunction in schizophrenia. In this mini review, I discuss the findings of monoamine PET studies, specifically focusing on neurocognitive functions in individuals with schizophrenia.

## PET techniques for evaluation of monoamine transmission

Most of the radiotracers used for neuroimaging-based quantification of receptors and transporters are pharmacologically antagonists that reversibly and specifically bind to these targets. The main outcome measure is the binding potential relative to the concentration of non-displaceable radiotracer in the brain (*BP*_ND_), corresponding to the ratio of the density of receptors or transporters available to bind radiotracer *in vivo* (*B*_avail_) to the dissociation constant of the radiotracer (*K*_D_) (10, 11). The gold standard of kinetic analysis of brain PET measurements is a compartment analysis with arterial input function, which requires arterial blood sampling and metabolite analysis of the parent compound. However, simplified methods can be applied if a reference region that is devoid of the receptor/transporter, such as the cerebellum, is present. These simplified methods are less invasive and more suitable for clinical use. For this review, I have selected PET studies performed under resting conditions (mainly for unmedicated individuals), which evaluate receptor/transporter availability and neurotransmitter synthesis capacity. Single photon emission computed tomography (SPECT) studies were not included because PET has a much higher sensitivity and spatial resolution than does SPECT, and more tracers have been developed for PET than for SPECT. The main PET tracers used for the measurement of central monoaminergic transmission are listed in Table 1. Some PET tracers have been reported to measure dopamine release, however, I did not include these challenge studies because my primary interest was to provide an overview of various monoamine transmissions in basic and simple conditions. A summary of studies that investigated both monoamine PET and cognitive functions in patients with schizophrenia and/or healthy subjects is listed in Table 2.

**Table 1:** PET probes used for the measurement of central monoaminergic transmission.

**Monoamine**	**Target**	**Name of PET probes**
Dopamine	D1	[^11^C]SCH23390, [^11^C]NNC112
	D2/D3	[^11^C]raclopride^*^, [^11^C]FLB457^*^, [^18^F]fallypride^*^
	Transporter	[^11^C]CFT
	Synthesis	[^18^F]DOPA, [^11^C]DOPA
Serotonin	1A	[^11^C]WAY100635
	2A	[^18^F]altanserin
	Transporter	[^11^C]DASB
Norepinephrine	Transporter	[^18^F]FMeNER-D2

* *These probes can be used to measure dopamine release*.

**Table 2:** Summary of studies that investigated both monoamine PET and cognitive functions in patients with schizophrenia and/or healthy subjects.

**Study**	**Year**	**Subjects**	**Cognitive tasks**	**PET probe**	**Findings**
Okubo et al. (20)	1997	17 with Sch and 18 HV	WCST	[^11^C]SCH23390	Reduced prefrontal D1RA was associated with poor WCST performance
Abi-Dargham et al. (21)	2002	16 with Sch and 16 HV	N-back	[^11^C]NNC112	Increased prefrontal D1RA was associated with poor working memory
Takahashi et al. (22)	2008	23 HV	WCST, ROCFT, RAVLT	[^11^C]SCH23390	An inverted U-shaped relationship between prefrontal D1RA and WCST performance
Hirvonen et al. (27)	2005	11 unaffected co-twins with Sch and 7 twin HV	WMS-R, CVLT	[^11^C]raclopride	Higher D2RA in the caudate was associated with a poor performance on tasks related to schizophrenia vulnerability
Cervenka et al. (29)	2008	16 HV	Pair associative learning, delayed pattern recognition memory, word recognition, WAIS-R, category fluency	[^11^C]raclopride	D2RA in the limbic striatum was related to episodic memory, D2RA in the associative and sensorimotor striatum showed associations with non-episodic tasks
Vyas et al. (35)	2017	25 with Sch and 19 HV	WCST, CVLT	[^18^F]fallypride	In individuals with Sch, D2RA was negatively correlated with WCST and CVLT performance whereas positive correlation was observed in HV
Takahashi et al. (36)	2007	25 HV	RAVLT, ROCFT, WCST	[^11^C]FLB457	Hippocampal D2RA was positively correlated with memory and also associated with frontal lobe functions
Yoder et al. (38)	2004	10 with Sch (most were medicated)	PANSS	[^11^C]CFT	Striatal DAT availability was inversely correlated with scores on the cognitive subscale of PANSS
Velnaleken et al. (48)	2007	11 HV	CPT, Stroop, TMT, WCST	[^18^F]DOPA	Positive correlations between DA synthesis capacity in the caudate nucleus, putamen, and midbrain with performance on TMT-B, CPT, and Stroop test
Meyer-Lindenberg et al. (41)	2002	6 with Sch and 6 HV	WCST	[^18^F]DOPA	Decreased PFC activation measured with fMRI predicted exaggerated striatal DA synthesis capacity
McGowan et al. (49)	2004	16 medicated individuals with Sch and 12 HV	Stroop, VF, SDMT	[^18^F]DOPA	Negative correlations between Stroop interference scores and DA synthesis capacity in the ACC in both individuals with Sch and HV
Howes et al. (43)	2009	24 prodromal individuals with Sch, 6 with Sch, and 12 HV	VF	[^18^F]DOPA	Within the prodromal Sch group, performance on the semantic VF task was negatively correlated with striatal DA synthesis capacity
Yasuno et al. (58)	2003	16 HV	WMS-R	[^11^C]WAY100635	Negative correlation between explicit memory function and 5-HT1ARA in hippocampus
Borg et al. (59)	2006	24 HV	Claeson–Dahl Learning and Memory Test, CPT, spatial working memory test, ROCFT, controlled oral assessment, WCST	[^11^C]WAY100635	No correlation between performance on any of the cognitive tests and 5-HT1ARA in the raphe, hippocampus, and neocortex
Penttila et al. (60)	2016	24 HV	WCST, WMS-R	[^11^C]WAY100635	Global 5-HT1ARA was positively correlated with verbal memory
Rasmussen et al. (61)	2010	30 with Sch and 30 HV	Spatial working memory, Stockings of Cambridge, Intra-Extradimensional set-shifting, rapid visual information processing	[^18^F]altanserin	No correlation between neurocognitive measures and 5-HT2ARA in any region
Madsen et al. (63)	2011	32 HV	Stroop, TMT, RAVLT, ROCFT, Intelligenz-Struktur-Test 2000 R	[^11^C]DASB	Positive associations between 5-HTT availability and Stroop test performance and logical reasoning. No association between 5-HTT availability and memory

*Sch, schizophrenia; HV, healthy volunteer; RA, receptor availability; WCST, Wisconsin Card Sorting Test; ROCFT, Rey-Osterrieth's Complex Figure Test; RAVLT, Rey's Auditory Verbal Learning Test; WMS-R, Wechsler Memory Scale-Revised; CVLT, California Verbal Learning Test; WAIS-R, Wechsler Adult Intelligent Scale-Revised; DLPFC, dorsolateral prefrontal cortex; PANSS, Positive and Negative Syndrome Scale; DAT, dopamine transporter; CPT, Continuous Performance Test; TMT, Trail Making Test; fMRI, functional magnetic resonance imaging; VF, verbal fluency; SDMT, Symbol-Digit Modality Test; ACC, anterior cingulate cortex; 5-HT1A, 5-hydroxytryptamine 1A; 5-HT2A, 5-hydroxytryptamine 2A; 5-HTT, 5-hydroxytryptamine transporter*.

## Dopamine

Dopamine is the main neurotransmitter involved in the pathophysiology and treatment of schizophrenia (12). Dopamine pathways have been well illustrated by PET with different radiotracers (13) and these PET tracers have been used to elucidate various aspects of aberrant dopaminergic transmission in schizophrenia for review see (14–16).

### D1 receptors

D1 receptors are densely localized in the striatum, and uniformly distributed in the neocortical regions (13). Evidence from animal and clinical research has suggested the presence of prefrontal dysfunctions in schizophrenia, and the D1 receptors in this brain region are considered to play crucial roles in various frontal lobe functions such as working memory (17–19).

Using the tracer [^11^C]SCH23390, Okubo et al. showed that patients with schizophrenia had lower D1 availability in the prefrontal cortex (20). They examined 17 male patients with schizophrenia (10 antipsychotic-naïve and 7 antipsychotic-free) and 18 healthy male controls. Availability of the D1 receptor in the prefrontal cortex was negatively correlated with the severity of negative symptoms, and was also associated with poor performance on the Wisconsin Card Sorting Test (WCST), which assesses executive function and prefrontal function. In contrast, using the tracer [^11^C]NNC112, Abi-Dargham et al. reported increased availability of the D1 receptor in the dorsolateral prefrontal cortex of 16 untreated patients with schizophrenia (7 antipsychotic-naïve and 9 antipsychotic-free) compared to that in 16 healthy subjects (21). The increased availability was related to n-back task performance, which represents working memory. The discrepancy in these results could arise owing to difference in the background of patients (age, sex, and prior exposure to antipsychotics) and the different tracers used. Additionally, the results of a study by Takahashi et al. provide a potential explanation for this discrepancy. Using [^11^C]SCH23390 PET in healthy subjects, they found a U-shaped relationship between prefrontal D1 receptor availability and the performance on the WCST, indicating that too little or too much D1 receptor stimulation hampers working memory or set shifting (22, 23), which was also hypothesized from the results of animal studies (18, 19).

### D2/D3 receptors

The striatum, which has dense dopamine innervation, is usually imaged using a moderate-affinity PET probe such as [^11^C]raclopride, and extrastriatal regions with very low levels of D2/D3 receptors, including the cortex, limbic regions, and thalamus, are imaged by high-affinity PET probes such as [^11^C]FLB457 and [^18^F]fallypride (Table 1). All these tracers are benzamide derivatives and antagonistically bind to both D2 and D3 receptors.

Dopamine D2 receptors are the primary target of currently available antipsychotic drugs owing to their potential to block these receptors (1, 2). Accordingly, several PET studies have investigated dopamine D2 receptors in schizophrenia beginning from the 1980's. However, previous reviews and meta-analyses PET and SPECT studies showed no difference or a small elevation in striatal D2/D3 receptor availability in unmedicated patients with schizophrenia compared to that in healthy controls under resting conditions (15, 24–26); however, the elevation was not evident in drug-naïve patients (26). Among these studies, a few evaluated cognitive functions because most studies focused on the psychopathology, particularly positive symptoms; therefore, less attention was given to cognitive impairment involving striatal dopamine D2 receptors in schizophrenia. Using PET and [^11^C]raclopride, Hirvonen and colleagues (27) examined 6 monozygotic and 5 dizygotic unaffected co-twins of patients with schizophrenia and 4 monozygotic and 3 dizygotic healthy control twins. They found that dopamine D2 receptor availability in the caudate was upregulated in unaffected monozygotic co-twins, and this upregulation was associated with poor performance on cognitive tasks such as a part of the Wechsler Memory Scale-Revised and the California Verbal Learning Test (CVLT).

The striatum can be divided into three functional subdivisions: the limbic, associative, and sensorimotor striatum (28). In healthy subject, using [^11^C]raclopride PET, Cervenka et al. found a distinct pattern of correlations among the striatal subregions; D2 receptor availability in the limbic striatum was related to performance on episodic memory, while that in the associative and sensorimotor striatum showed associations to non-episodic tasks (29).

With regard to extrastriatal D2/D3 receptors, significant differences in their availability between schizophrenia and healthy controls have been reported particularly in the thalamus (30–33); a meta-analysis found the summary effect size for thalamic D2/D3 availability was *d* = −0.32, however, did not reach significance (34). In a very recent comprehensive study, Vyas et al. used [^18^F]fallypride to evaluate executive dysfunction and memory impairment in patients with schizophrenia (35). Twenty medication-naïve and 5 drug-free patients with schizophrenia underwent the WCST and CVLT. Patients with schizophrenia showed negative or low correlations between D2/D3 receptor availability and WCST performance, while healthy subjects showed positive correlations, suggesting better performance with higher D2/D3 receptor availability; the difference was marked in the thalamus. Similarly, patients showed negative or very low correlations between D2/D3 receptor availability in the fronto-striatal-thalamic regions and performance on the CVLT, while healthy subjects showed a positive correlation. In another study in healthy subjects, Takahashi et al. reported a relationship between hippocampal dopamine D2 receptors and not only memory but also frontal functions such as executive functions and verbal fluency (36).

### Dopamine transporter (DAT)

The majority of molecular imaging studies investigating striatal DAT availability failed to find any significant differences between healthy controls and untreated patients with schizophrenia (15), and this finding was supported by the results of recent meta-analyses that included PET and SPECT studies (26, 37). One study showed that schizophrenia patients with tardive dyskinesia had lower DAT availability than schizophrenia patients without tardive dyskinesia, and that striatal DAT availability was correlated with the severity of negative symptoms, and cognitive and depression/anxiety scores on the positive and negative syndrome scale (38). However, most of the subjects in the study were medicated and the medication effect needs to be considered. In addition, no MRI scans were available for all patients, and positive and negative syndrome scale scores were not assessed for some patients. To the best of my knowledge, measures of cognitive functions were not evaluated in any other PET studies on DAT in schizophrenia.

### L-DOPA uptake (dopamine synthesis capacity)

The endogenous dopamine synthesis rate is commonly measured using 6-[^18^F]fluoro-L-DOPA or L-[β-^11^C]DOPA, two radioactive analogs of the dopamine precursor L-DOPA, which are indicative of dopamine synthesis capacity in presynaptic terminals (13, 16). In schizophrenia compared to healthy controls, increased dopamine synthesis capacity has been consistently shown in the majority of previous studies (39–46) (for review see (15, 16)) and recent meta-analyses confirmed the findings with large effect sizes (26, 47).

In a study investigating the association between human cognitive function and dopamine synthesis capacity, Velnaleken et al. (48) found significant positive correlations in healthy subjects between the dopamine synthesis capacity in the striatum and performance on the trail-making test-B, continuous performance test, and Stroop test. In one study with unmedicated schizophrenia patients (41), PET with [^18^F]DOPA and [^15^O]H_2_O (which measures cerebral blood flow) revealed that patients with schizophrenia had higher dopamine synthesis capacity in the striatum than healthy controls did, indicating exaggerated presynaptic dopamine function. In patients with schizophrenia, the increase in cerebral blood flow in the dorsolateral prefrontal cortex during the WCST task was tightly coupled with striatal dopamine synthesis capacity; this relationship was not found in healthy controls. In another study, [^18^F]DOPA PET in patients with schizophrenia on antipsychotic medication revealed that the dopamine synthesis capacity in the dorsal anterior cingulate was correlated with performance on the Stroop Color-Word Test (49).

During the manifestation of prodromal symptoms of schizophrenia before the onset of psychosis, patients showed elevated striatal dopamine synthesis capacity in the associative striatum (43). In that study, the at-risk mental state group showed a negative correlation between the dopamine synthesis capacity in the associative striatum and the performance on the semantic verbal fluency task, i.e., greater elevation in synthesis was associated with fewer correct responses and a similar negative correlation was observed for phonologic verbal fluency.

## Serotonin (5-hydroxytryptamine, 5-HT)

In the human brain, the serotonergic system has 14 diverse receptor subtypes and transporters (50). Because of the availability of suitable radiotracers, extensive PET studies have been performed to investigate the availability of 5-HT1A and 5-HT2A receptors and the 5-HT transporter in various neuropsychiatric disorders. These receptors and transporters are of interest because they are main targets of pharmacotherapy via psychotropic drugs (51, 52). However, the number of imaging studies investigating the central serotonergic system in schizophrenia is limited (53).

### 5-HT1A receptors

5-HT1A receptors are widely distributed in the hippocampal regions, insula, neocortical regions, and dorsal raphe nucleus (54). In addition, because the 5-HT1A receptor modulates the entire serotonin system, it is one of the most important 5-HT receptor subtypes (55). Several lines of evidence from animal studies and pharmacological studies indicate that the 5-HT1A receptor plays an important role in cognitive function and is a promising target for the treatment of cognitive and affective symptoms in neuropsychiatric disorders, including schizophrenia (51, 52, 55).

Thus far, four studies have examined 5-HT1A availability in schizophrenia, using the same PET tracer [^11^C]WAY100635. One study reported an increase in 5-HT1A availability in the medial temporal cortex of schizophrenia patients (56), whereas another reported a decrease in the amygdala (57); the remaining two reported no difference in 5-HT1A availability between schizophrenia patients and healthy controls, although a meta-analysis of postmortem studies found an elevation in prefrontal 5-HT1A in schizophrenia (53).

With regard to cognitive function in healthy subjects, Yasuno et al. found a negative correlation between explicit memory function and 5-HT1A receptor availability in the hippocampus (58), while Borg et al., who performed [^11^C]WAY100635 PET and used the same simplified reference model, found no correlation between regional 5-HT1A receptor availability in the raphe nuclei, hippocampus, and neocortex and various domains of cognitive performance (55, 59). However, a recent study by Penttila et al. found that global 5-HT1A receptor binding, measured with the gold standard method based on kinetic modeling using arterial blood samples, was positively correlated with measures of verbal memory in healthy subjects (60). To date, no 5-HT1A PET data have been reported for patients with schizophrenia in relation to cognitive function.

### 5-HT2A receptor

A large body of evidence from postmortem and pharmacological studies suggests that 5-HT2A receptors play an important role in schizophrenia and cognition (51, 52). A meta-analysis of postmortem studies found a reduction in prefrontal 5-HT2A receptors in patients with schizophrenia (53). However, only a few PET studies have been performed on first-episode antipsychotic-naïve patients with schizophrenia, and their results are inconsistent (53). In a recent study with the largest sample size to date, a total of 30 patients with schizophrenia and matched healthy controls underwent [^18^F]altanserin PET scans, which are highly selective for 5-HT2A receptors (61). Patients with schizophrenia showed lower 5-HT2A availability in the frontal cortex than healthy controls did. However, no correlations were found between 5-HT2A availability and cognitive functions such as working memory, attention, and executive functions, suggesting that 5-HT2A receptors are not involved in cognitive dysfunction, at least in the early stage of schizophrenia.

### Serotonin transporter (5-HTT)

A previous PET study with the 5-HTT-selective tracer [^11^C]DASB found no significant difference in 5-HTT availability between patients with schizophrenia and healthy control subjects, and no correlation between 5-HTT availability and schizophrenia symptoms (62). In contrast, another [^11^C]DASB PET study with healthy subjects found that 5-HTT availability in the fronto-striatal regions was associated with better performance on executive function and logical reasoning (63). To date, however, no PET studies have investigated the association between 5-HTT availability and cognitive function in patients with schizophrenia.

### Other serotonergic system

PET probes for 5-HT1B, 5HT4, 5-HT6, and 5-HT synthesis have been successfully used for human brain imaging (50); however, to date, no clinical studies have used these probes for imaging of the brains of patients with schizophrenia.

## Norepinephrine

The central norepinephrine system plays crucial roles in arousal and concentration, and the norepinephrine transporter is a target in pharmacotherapy for depression and attention-deficit hyperactivity disorder. Despite this, few PET probes have been developed for norepinephrine transporter (NET) imaging (64). To date, there have been no reports of PET imaging of the NET in patients with schizophrenia.

## Discussion and future directions

PET provides a direct way of investigating the neurotransmission and neurobiology of schizophrenia. Numerous PET studies have revealed differences between the brains of patients with schizophrenia and those of healthy controls, and the association of these brain changes with symptom scales, suggesting the underpinnings of the pathophysiology of this disorder. As reviewed above, however, only a limited number of PET studies have directly investigated the relationship between cognitive dysfunctions, assessed using neuropsychological tests, and monoamine transmission *in vivo*, and found significant associations between them. Consequently, dysregulated striatal dopamine synthesis capacity, and prefrontal D1 receptor and extrastriatal D2/D3 receptor availability might be at least partly indicative of cognitive impairment in schizophrenia. However, studies are scarce and not systematic: differences in radiotracers used, methods of quantification, neurocognitive tests employed, and patient characteristics (such as phase of the illness, prior exposure to antipsychotics, age, and sex) could be confounders. This scarcity and lack of systematic approaches occurs largely because it is difficult to recruit many patients with unmedicated schizophrenia; therefore, multi-site studies with common protocols are needed. Moreover, different approaches such as measuring endogenous release of transmitters rather than focusing only on baseline receptor availability, could be useful for precise evaluation of neurotransmission (65, 66). In summary, pharmacological challenge techniques such as that with amphetamine and dopamine depletion using [^11^C]raclopride, which reflect presynaptic dopamine function, have been well replicated to detect dysregulated dopamine neurotransmission as a pathophysiology of schizophrenia (14, 67). Another approach is to measure changes in receptor binding of a radiotracer while performing a cognitive task as it accurately reflects the amount of neurotransmitter released during the task (68–70). Furthermore using agonist tracers such as [^11^C]-(+)-PHNO and [^11^C]MNPA, that can be more sensitive to endogenous transmitters, might help detect high affinity states of dopamine D2 receptors that could be more responsible for the pathophysiology based on a hypothesis from *in vitro* studies (71). Although studies on 5-HT1A receptors and 5-HT2A receptors have not demonstrated their involvement in cognition in schizophrenia, newer serotonergic targets, such as 5-HT4, might potentially be associated with cognitive functions (50). Regarding the norepinephrine system, one study reported a relationship between attention function and NET availability in patients with depression (72), which might be applicable to some patients with schizophrenia. Moreover, other neurotransmitter systems such as glutaminergic or cholinergic systems might also be involved in cognitive impairment in schizophrenia. Further development of optimal PET tracers and new techniques to measure more precise neurotransmission in human PET imaging will also provide new insights to the neurobiology of cognitive dysfunction in schizophrenia.

## Author contributions

The author confirms being the sole contributor of this work and approved it for publication.

## Conflict of interest statement

The author declares that the research was conducted in the absence of any commercial or financial relationships that could be construed as a potential conflict of interest.

## References

[1] Zipursky RB Meyer JH Verhoeff NP. PET and SPECT imaging in psychiatric disorders. Can J Psychiatry (2007) 52:146–57. 10.1177/070674370705200303 17479522

[2] van Os J Kapur S. Schizophrenia. Lancet (2009) 374:635–45. 10.1016/S0140-6736(09)60995-8 19700006

[3] Fioravanti M Carlone O Vitale B Cinti ME Clare L. A meta-analysis of cognitive deficits in adults with a diagnosis of schizophrenia. Neuropsychol Rev. (2005) 15:73–95. 10.1007/s11065-005-6254-9 16211467

[4] Szoke A Trandafir A Dupont ME Meary A Schurhoff F Leboyer M. Longitudinal studies of cognition in schizophrenia: meta-analysis. Br J Psychiatry (2008) 192:248–57. 10.1192/bjp.bp.106.029009 18378982

[5] Reichenberg A Harvey PD. Neuropsychological impairments in schizophrenia: integration of performance-based and brain imaging findings. Psychol Bull. (2007) 133:833–58. 10.1037/0033-2909.133.5.833 17723032

[6] Kircher TT Thienel R. Functional brain imaging of symptoms and cognition in schizophrenia. Prog Brain Res. (2005) 150:299–308. 10.1016/S0079-6123(05)50022-0 16186032

[7] Ragland JD Laird AR Ranganath C Blumenfeld RS Gonzales SM Glahn DC. Prefrontal activation deficits during episodic memory in schizophrenia. Am J Psychiatry (2009) 166:863–74. 10.1176/appi.ajp.2009.08091307 19411370 PMC2885958

[8] Minzenberg MJ Laird AR Thelen S Carter CS Glahn DC. Meta-analysis of 41 functional neuroimaging studies of executive function in schizophrenia. Arch Gen Psychiatry (2009) 66:811–22. 10.1001/archgenpsychiatry.2009.91 19652121 PMC2888482

[9] Carter CS Barch DM. Imaging biomarkers for treatment development for impaired cognition: report of the sixth CNTRICS meeting: biomarkers recommended for further development. Schizophr Bull. (2012) 38:26–33. 10.1093/schbul/sbr109 21914642 PMC3245593

[10] Ito H Naganawa M Seki C Takano H Kanno I Suhara T Quantification of Neuroreceptors and Neurotransporters. In: Gründer G editor. Molecular Imaging in the Clinical Neurosciences. 71 New York, NY: Springer (2012). p. 149–61.

[11] Innis RB Cunningham VJ Delforge J Fujita M Gjedde A Gunn RN. Consensus nomenclature for *in vivo* imaging of reversibly binding radioligands. J Cereb Blood Flow Metab. (2007) 27:1533–9. 10.1038/sj.jcbfm.9600493 17519979

[12] Howes OD Kapur S. The dopamine hypothesis of schizophrenia: version III–the final common pathway. Schizophr Bull. (2009) 35:549–62. 10.1093/schbul/sbp006 19325164 PMC2669582

[13] Ito H Takahashi H Arakawa R Takano H Suhara T. Normal database of dopaminergic neurotransmission system in human brain measured by positron emission tomography. Neuroimage (2008) 39:555–65. 10.1016/j.neuroimage.2007.09.011 17962043

[14] Weinstein JJ Chohan MO Slifstein M Kegeles LS Moore H Abi-Dargham A. Pathway-specific dopamine abnormalities in schizophrenia. Biol Psychiatry (2017) 81:31–42. 10.1016/j.biopsych.2016.03.2104 27206569 PMC5177794

[15] Dean B. Neurochemistry of schizophrenia: the contribution of neuroimaging postmortem pathology and neurochemistry in schizophrenia. Curr Top Med Chem. (2012) 12:2375–92. 10.2174/156802612805289935 23279177

[16] Brunelin J Fecteau S Suaud-Chagny MF. Abnormal striatal dopamine transmission in schizophrenia. Curr Med Chem. (2013) 20:397–404. 23157632 10.2174/0929867311320030011 PMC3866953

[17] Goldman-Rakic PS Selemon LD. Functional and anatomical aspects of prefrontal pathology in schizophrenia. Schizophr Bull. (1997) 23:437–58. 10.1093/schbul/23.3.437 9327508

[18] Goldman-Rakic PS Muly EC III Williams GV. D1 receptors in prefrontal cells and circuits. Brain Res Brain Res Rev. (2000) 31:295–301. 10.1016/S0165-0173(99)00045-4 10719156

[19] Seamans JK Yang CR. The principal features and mechanisms of dopamine modulation in the prefrontal cortex. Prog Neurobiol. (2004) 74:1–58. 10.1016/j.pneurobio.2004.05.006 15381316

[20] Okubo Y Suhara T Suzuki K Kobayashi K Inoue O Terasaki O. Decreased prefrontal dopamine D1 receptors in schizophrenia revealed by PET. Nature (1997) 385:634–6. 10.1038/385634a0 9024661

[21] Abi-Dargham A Mawlawi O Lombardo I Gil R Martinez D Huang Y. Prefrontal dopamine D1 receptors and working memory in schizophrenia. J Neurosci. (2002) 22:3708–19. 10.1523/JNEUROSCI.22-09-03708.2002 11978847 PMC6758376

[22] Takahashi H Kato M Takano H Arakawa R Okumura M Otsuka T. Differential contributions of prefrontal and hippocampal dopamine D1 and D2 receptors in human cognitive functions. J Neurosci. (2008) 28:12032–8. 10.1523/JNEUROSCI.3446-08.2008 19005068 PMC6671660

[23] Takahashi H. PET neuroimaging of extrastriatal dopamine receptors and prefrontal cortex functions. J Physiol Paris (2013) 107:503–9. 10.1016/j.jphysparis.2013.07.001 23851135

[24] Salavati B Rajji TK Price R Sun Y Graff-Guerrero A Daskalakis ZJ. Imaging-based neurochemistry in schizophrenia: a systematic review and implications for dysfunctional long-term potentiation. Schizophr Bull. (2015) 41:44–56. 10.1093/schbul/sbu132 25249654 PMC4266301

[25] Laruelle M. Imaging dopamine transmission in schizophrenia. A review and meta-analysis. Q J Nucl Med. (1998) 42:211–21. 9796369

[26] Howes OD Kambeitz J Kim E Stahl D Slifstein M Abi-Dargham A. The nature of dopamine dysfunction in schizophrenia and what this means for treatment. Arch Gen Psychiatry (2012) 69:776–86. 10.1001/archgenpsychiatry.2012.169 22474070 PMC3730746

[27] Hirvonen J van Erp TG Huttunen J Aalto S Nagren K Huttunen M. Increased caudate dopamine D2 receptor availability as a genetic marker for schizophrenia. Arch Gen Psychiatry (2005) 62:371–8. 10.1001/archpsyc.62.4.371 15809404

[28] Mawlawi O Martinez D Slifstein M Broft A Chatterjee R Hwang DR. Imaging human mesolimbic dopamine transmission with positron emission tomography: I. Accuracy and precision of D2 receptor parameter measurements in ventral striatum. J Cereb Blood Flow Metab. (2001) 21:1034–57. 10.1097/00004647-200109000-00002 11524609

[29] Cervenka S Backman L Cselenyi Z Halldin C Farde L. Associations between dopamine D2-receptor binding and cognitive performance indicate functional compartmentalization of the human striatum. Neuroimage (2008) 40:1287–95. 10.1016/j.neuroimage.2007.12.063 18296072

[30] Yasuno F Suhara T Okubo Y Sudo Y Inoue M Ichimiya T. Low dopamine D2 receptor binding in subregions of the thalamus in schizophrenia. Am J Psychiatry (2004) 161:1016–22. 10.1176/appi.ajp.161.6.1016 15169689

[31] Kessler RM Woodward ND Riccardi P Li R Ansari MS Anderson S. Dopamine D2 receptor levels in striatum, thalamus, substantia nigra, limbic regions, and cortex in schizophrenic subjects. Biol Psychiatry (2009) 65:1024–31. 10.1016/j.biopsych.2008.12.029 19251247 PMC2951678

[32] Takahashi H Higuchi M Suhara T. The role of extrastriatal dopamine D2 receptors in schizophrenia. Biol Psychiatry (2006) 59:919–28. 10.1016/j.biopsych.2006.01.022 16682269

[33] Lehrer DS Christian BT Kirbas C Chiang M Sidhu S Short H. ^18^F-fallypride binding potential in patients with schizophrenia compared to healthy controls. Schizophr Res. (2010) 122:43–52. 10.1016/j.schres.2010.03.043 20655709 PMC3278159

[34] Kambeitz J Abi-Dargham A Kapur S Howes OD. Alterations in cortical and extrastriatal subcortical dopamine function in schizophrenia: systematic review and meta-analysis of imaging studies. Br J Psychiatry (2014) 204:420–9. 10.1192/bjp.bp.113.132308 25029687

[35] Vyas NS Buchsbaum MS Lehrer DS Merrill BM DeCastro A Doninger NA. D2/D3 dopamine receptor binding with [^18^F]fallypride correlates of executive function in medication-naive patients with schizophrenia. Schizophr Res. (2017) 192:442–56. 10.1016/j.schres.2017.05.017 28576546

[36] Takahashi H Kato M Hayashi M Okubo Y Takano A Ito H. Memory and frontal lobe functions; possible relations with dopamine D2 receptors in the hippocampus. Neuroimage (2007) 34:1643–9. 10.1016/j.neuroimage.2006.11.008 17174573

[37] Fusar-Poli P Meyer-Lindenberg A. Striatal presynaptic dopamine in schizophrenia, Part I: meta-analysis of dopamine active transporter (DAT) density. Schizophr Bull. (2013) 39:22–32. 10.1093/schbul/sbr111 22282456 PMC3523907

[38] Yoder KK Hutchins GD Morris ED Brashear A Wang C Shekhar A. Dopamine transporter density in schizophrenic subjects with and without tardive dyskinesia. Schizophr Res. (2004) 71:371–5. 10.1016/j.schres.2004.03.015 15474908

[39] Hietala J Syvalahti E Vuorio K Rakkolainen V Bergman J Haaparanta M. Presynaptic dopamine function in striatum of neuroleptic-naive schizophrenic patients. Lancet (1995) 346:1130–1. 10.1016/S0140-6736(95)91801-9 7475604

[40] Lindstrom LH Gefvert O Hagberg G Lundberg T Bergstrom M Hartvig P. Increased dopamine synthesis rate in medial prefrontal cortex and striatum in schizophrenia indicated by L-(beta-^11^C) DOPA and PET. Biol Psychiatry (1999) 46:681–8. 10.1016/S0006-3223(99)00109-2 10472420

[41] Meyer-Lindenberg A Miletich RS Kohn PD Esposito G Carson RE Quarantelli M. Reduced prefrontal activity predicts exaggerated striatal dopaminergic function in schizophrenia. Nat Neurosci. (2002) 5:267–71. 10.1038/nn804 11865311

[42] Nozaki S Kato M Takano H Ito H Takahashi H Arakawa R. Regional dopamine synthesis in patients with schizophrenia using L-[beta-^11^C]DOPA PET. Schizophr Res. (2009) 108:78–84. 10.1016/j.schres.2008.11.006 19056247

[43] Howes OD Montgomery AJ Asselin MC Murray RM Valli I Tabraham P. Elevated striatal dopamine function linked to prodromal signs of schizophrenia. Arch Gen Psychiatry (2009) 66:13–20. 10.1001/archgenpsychiatry.2008.514 19124684

[44] Kumakura Y Cumming P Vernaleken I Buchholz H-G Siessmeier T Heinz A. Elevated [^18^F]fluorodopamine turnover in brain of patients with schizophrenia: an [^18^F]fluorodopa/positron emission tomography study. J Neurosci. (2007) 27:8080–7. 10.1523/JNEUROSCI.0805-07.2007 17652599 PMC6672729

[45] Dao-Castellana MH Paillere-Martinot ML Hantraye P Attar-Levy D Remy P Crouzel C. Presynaptic dopaminergic function in the striatum of schizophrenic patients. Schizophr Res. (1997) 23:167–74. 10.1016/S0920-9964(96)00102-8 9061812

[46] Elkashef AM Doudet D Bryant T Cohen RM Li SH Wyatt RJ. 6-^18^F-DOPA PET study in patients with schizophrenia. Positron emission tomography. Psychiatry Res. (2000) 100:1–11. 10.1016/S0925-4927(00)00064-0 11090720

[47] Fusar-Poli P Meyer-Lindenberg A. Striatal presynaptic dopamine in schizophrenia, part II: meta-analysis of ^[18^F/^11^C]-DOPA PET studies. Schizophr Bull. (2013) 39:33–42. 10.1093/schbul/sbr180 22282454 PMC3523905

[48] Vernaleken I Buchholz HG Kumakura Y Siessmeier T Stoeter P Bartenstein P. 'Prefrontal' cognitive performance of healthy subjects positively correlates with cerebral FDOPA influx: an exploratory [18F]-fluoro-L-DOPA-PET investigation. Hum Brain Mapp. (2007) 28:931–9. 10.1002/hbm.20325 17133402 PMC6871482

[49] McGowan S Lawrence AD Sales T Quested D Grasby P. Presynaptic dopaminergic dysfunction in schizophrenia: a positron emission tomographic [^18^F]fluorodopa study. Arch Gen Psychiatry (2004) 61:134–42. 10.1001/archpsyc.61.2.134 14757589

[50] Paterson LM Kornum BR Nutt DJ Pike VW Knudsen GM. 5-HT radioligands for human brain imaging with PET and SPECT. Med Res Rev. (2013) 33:54–111. 10.1002/med.20245 21674551 PMC4188513

[51] Roth BL Hanizavareh SM Blum AE. Serotonin receptors represent highly favorable molecular targets for cognitive enhancement in schizophrenia and other disorders. Psychopharmacology (Berl) (2004) 174:17–24. 10.1007/s00213-003-1683-8 15205874

[52] Meltzer HY Li Z Kaneda Y Ichikawa J. Serotonin receptors: their key role in drugs to treat schizophrenia. Prog Neuropsychopharmacol Biol Psychiatry (2003) 27:1159–72. 10.1016/j.pnpbp.2003.09.010 14642974

[53] Selvaraj S Arnone D Cappai A Howes O. Alterations in the serotonin system in schizophrenia: a systematic review and meta-analysis of postmortem and molecular imaging studies. Neurosci Biobehav Rev. (2014) 45:233–45. 10.1016/j.neubiorev.2014.06.005 24971825

[54] Takano H Ito H Takahashi H Arakawa R Okumura M Kodaka F Serotonergic neurotransmission in the living human brain: a positron emission tomography study using [^11^C]DASB and [^11^C]WAY100635 in young healthy men. Synapse (2010) 65:624–33. 10.1002/syn.20883 21484882

[55] Borg J. Molecular imaging of the 5-HT1A receptor in relation to human cognition. Behav Brain Res. (2008) 195:103–11. 10.1016/j.bbr.2008.06.011 18606193

[56] Tauscher J Kapur S Verhoeff NP Hussey DF Daskalakis ZJ Tauscher-Wisniewski S. Brain serotonin 5-HT1A receptor binding in schizophrenia measured by positron emission tomography and [^11^C]WAY-100635. Arch Gen Psychiatry (2002) 59:514–20. 10.1001/archpsyc.59.6.514 12044193

[57] Yasuno F Suhara T Ichimiya T Takano A Ando T Okubo Y. Decreased 5-HT1A receptor binding in amygdala of schizophrenia. Biol Psychiatry (2004) 55:439–44. 10.1016/j.biopsych.2003.11.016 15023569

[58] Yasuno F Suhara T Nakayama T Ichimiya T Okubo Y Takano A. Inhibitory effect of hippocampal 5-HT1A receptors on human explicit memory. Am J Psychiatry (2003) 160:334–40. 10.1176/appi.ajp.160.2.334 12562581

[59] Borg J Andree B Lundberg J Halldin C Farde L. Search for correlations between serotonin 5-HT1A receptor expression and cognitive functions–a strategy in translational psychopharmacology. Psychopharmacology (Berl) (2006) 185:389–94. 10.1007/s00213-006-0329-z 16541245

[60] Penttila J Hirvonen J Tuominen L Lumme V Ilonen T Nagren K. Verbal memory and 5-HT1A receptors in healthy volunteers–a PET study with [carbonyl-^11^C]WAY-100635. Eur Neuropsychopharmacol. (2016) 26:570–7. 10.1016/j.euroneuro.2015.12.028 26775837

[61] Rasmussen H Erritzoe D Andersen R Ebdrup BH Aggernaes B Oranje B. Decreased frontal serotonin2A receptor binding in antipsychotic-naive patients with first-episode schizophrenia. Arch Gen Psychiatry (2010) 67:9–16. 10.1001/archgenpsychiatry.2009.176 20048218

[62] Frankle WG Narendran R Huang Y Hwang DR Lombardo I Cangiano C. Serotonin transporter availability in patients with schizophrenia: a positron emission tomography imaging study with [^11^C]DASB. Biol Psychiatry (2005) 57:1510–6. 10.1016/j.biopsych.2005.02.028 15953487

[63] Madsen K Erritzoe D Mortensen EL Gade A Madsen J Baare W. Cognitive function is related to fronto-striatal serotonin transporter levels–a brain PET study in young healthy subjects. Psychopharmacology (Berl) (2011) 213:573–81. 10.1007/s00213-010-1926-4 20623110

[64] Schou M Pike VW Halldin C. Development of radioligands for imaging of brain norepinephrine transporters *in vivo* with positron emission tomography. Curr Top Med Chem. (2007) 7:1806–16. 10.2174/156802607782507411 17979789

[65] Laruelle M. Imaging synaptic neurotransmission with *in vivo* binding competition techniques: a critical review. J Cereb Blood Flow Metab. (2000) 20:423–51. 10.1097/00004647-200003000-00001 10724107

[66] Paterson LM Tyacke RJ Nutt DJ Knudsen GM. Measuring endogenous 5-HT release by emission tomography: promises and pitfalls. J Cereb Blood Flow Metab. (2010) 30:1682–706. 10.1038/jcbfm.2010.104 20664611 PMC3023404

[67] Slifstein M Abi-Dargham A. Recent developments in molecular brain imaging of neuropsychiatric disorders. Semin Nucl Med. (2017) 47:54–63. 10.1053/j.semnuclmed.2016.09.002 27987558

[68] Aalto S Bruck A Laine M Nagren K Rinne JO. Frontal and temporal dopamine release during working memory and attention tasks in healthy humans: a positron emission tomography study using the high-affinity dopamine D2 receptor ligand [^11^C]FLB 457. J Neurosci. (2005) 25:2471–7. 10.1523/JNEUROSCI.2097-04.2005 15758155 PMC6725173

[69] Badgaiyan RD Wack D. Evidence of dopaminergic processing of executive inhibition. PLoS ONE (2011) 6:e28075. 10.1371/journal.pone.0028075 22162756 PMC3230601

[70] Mizrahi R Addington J Rusjan PM Suridjan I Ng A Boileau I. Increased stress-induced dopamine release in psychosis. Biol Psychiatry (2012) 71:561–7. 10.1016/j.biopsych.2011.10.009 22133268

[71] Seeman P. Schizophrenia and dopamine receptors. Eur Neuropsychopharmacol. (2013) 23:999–1009. 10.1016/j.euroneuro.2013.06.005 23860356

[72] Moriguchi S Yamada M Takano H Nagashima T Takahata K Yokokawa K. Norepinephrine transporter in major depressive disorder: a PET study. Am J Psychiatry (2016):appiajp201615101334. 10.1176/appi.ajp.2016.15101334 27631962

